# How do we best relax control measures as vaccine coverage for SARS-CoV-2 rises?

**DOI:** 10.1016/j.lanwpc.2021.100271

**Published:** 2021-09-17

**Authors:** Brody H Foy, Brian Wahl

**Affiliations:** 1 Center for Systems Biology, Massachusetts General Hospital and Harvard Medical School, Boston, MA, USA, 02114.; 2 Department of International Health, Johns Hopkins Bloomberg School of Public Health, Baltimore, MD, USA, 21205

The COVID-19 pandemic has significantly altered movement and behaviour patterns of populations across the world. To reduce disease spread, countries have enacted varying levels of control measures, such as border restrictions, mask mandates, and social distancing policies.^1^ Given the rising rates of SARS-CoV-2 vaccination, a key challenge for each country is to determine the optimal speed to relax control measures at, as domestic vaccine coverage increases. In *The Lancet Regional Health – Western Pacific*, Trung Nguyen and colleagues^2^ use mathematical modelling to analyse how different vaccination strategies interact with control measures to affect COVID-19 transmission in New Zealand (NZ).

NZ faces unique considerations in relaxing control measure due to its extremely effective pursuance of a “zero-COVID policy”.^3^ Throughout the pandemic, NZ has used strong applications of border closure policies and lockdowns to maintain long periods without community spread. This has resulted in incredibly successful disease mitigation, with New Zealand being one of the only high-income countries to achieve a negative excess death rate in 2020.^4^ However, this also means that NZ has low rates of infection-induced immunity, increasing the vaccine coverage required to achieve broad population-level protection and potentially altering future transmission dynamics.^5^ Given this, there is a strong need to model how reopening the NZ border might affect domestic disease transmission – motivating Nguyen *et al*.’s work.

The core dynamics of disease spread within a population are typically modelled using well-established compartmental models. To account for country-specific factors, these models often account for the age-stratification of the population and age-specific contact mixing patterns. Nguyen et al. used a model of this class to simulate how age-structured vaccine allocation programs (i.e., prioritized vaccination of certain age groups) would influence the herd immunity threshold, assuming that international arrivals lead to a low number of imported cases each day, reflecting a relaxation of border control measures. In line with findings in other countries^5^^,^^6^ and with NZ's own vaccine allocation policy,^7^ they found that targeting vaccination to groups with the highest risk of mortality, including indigenous populations, was the most effective strategy in reducing deaths and hospital burden.

Their analyses suggest that if vaccines maintain an efficacy of 90% and 80% against symptomatic and asymptomatic infection respectively, reaching the herd immunity threshold may require more than 85% vaccine coverage in the general population. Given the current lack of vaccine availability for children under 12 years (∼15-20% of NZ's population), this threshold may be unachievable through vaccination alone. This implies that some degree of community spread might be inevitable upon the international border reopening and/or relaxation of international quarantine policies, unless other policies (e.g., social distancing, mask mandates) significantly reduce the effective transmissibility of the disease. The results do suggest, however, that high coverage among the most vulnerable populations will greatly reduce death rates and hospitalizations, even if the herd immunity threshold isn't achieved through vaccination alone.

The assumptions of vaccine efficacy against symptomatic and asymptomatic infection could be optimistic given the emerging evidence related to immune escape potential and higher viral load, even among breakthrough cases, of SARS-CoV-2 variants, especially the delta variant.^8^ Nevertheless, this work represents a good first step at quantifying the potential ramifications of NZ border reopening strategies. Further work should be undertaken to ascertain how the disease burden driven by border reopening may be unevenly distributed across populations – in particular the indigenous Maori population – due to differences in age structure, contact mixing patterns, and socioeconomic factors.^9^

Another complicating factor for modelling NZ border reopening is that vaccine uptake and infection-induced immunity may be correlated. Due to heavy politicisation of COVID control measures,^10^ vaccine-hesitant populations may be less likely to adhere to social distancing policies and thus more likely to become infected and attain some degree of natural immunity. This potential correlation may confound estimates of vaccine effectiveness and disease transmissibility in countries with high community spread. This is a common challenge of compartmental models and is difficult to address without implementing a cumbersome set of compartments.

Given the economic and social disruption that control measures may cause, it is not economically or politically feasible to maintain these measures indefinitely. However, the effect of vaccination on disease spread is non-linear, meaning reducing spread while uptake continues to steadily increase may significantly reduce overall hospital and death burden for NZ. Nguyen et al.’s analysis suggests that – barring currently unprecedented vaccine uptake – vaccination alone may not allow NZ to reach the herd immunity threshold. However, effective targeting of the vaccines to high-risk populations can achieve similar benefit. This further highlights the need for strong governmental policy to increase vaccine uptake and achieve as high a total population coverage as possible.

## Declaration of competing interest

The authors declare no conflict of interest
